# OTME-13. Integration of metabolic and transcriptional signatures of glioblastoma invasion reveals extracellular matrix reorganization and vasculature remodeling

**DOI:** 10.1093/noajnl/vdab070.064

**Published:** 2021-07-05

**Authors:** Cristina Cudalbu, Pierre Bady, Marta Lai, Lijing Xin, Olga Gusyatiner, Marie-France Hamou, Mario Lepore, Jean-Philippe Brouland, Roy Daniel, Andreas Hottinger, Monika Hegi

**Affiliations:** 1 CIBM Center for Biomedical Imaging, Lausanne, Switzerland; 2 Ecole Polytechnique Fédérale de Lausanne (EPFL), Lausanne, Switzerland; 3 SIB Swiss Center for Bioinformatics, Lausanne, Switzerland; 4 Lausanne University Hospital & University of Lausanne, Lausanne, Switzerland; 5 Swiss Cancer Center Léman, Lausanne, Switzerland

## Abstract

**Background:**

The invasive behavior of glioblastoma is considered highly relevant for recurrence. However, the invasion zone is difficult to visualize, typically lies outside the resected and irradiated area, and is protected by the blood brain barrier, posing a particular challenge for treatment. We present biological features of invasive growth accompanying tumor progression and invasion based on associated metabolic and transcriptomic changes in patient derived orthotopic xenografts (PDOX) and corresponding patients.

**Methods:**

Patients with suspected glioblastoma were enrolled (NCT02904525) and underwent ^1^H-MR spectroscopy and imaging (^1^H-MRS/I, 7T). Tissue obtained at surgery was transplanted orthotopically into immune-compromised mice. Longitudinal follow-up was performed by ^1^H-MRS/I (14.1T) on the injected and the contralateral side. The PDOX, the corresponding contralateral side, and the original human tumors underwent RNA-sequencing.

**Results:**

The temporal changes of the metabolite profiles characterized the kinetics of invasive growth of PDOX, and were patient specific. Comparison of ^1^H-MRS derived metabolite signatures, reflecting temporal changes of tumor development and invasion in PDOX, revealed high similarity to spatial metabolite signatures of combined multi-voxel analyses of the patients’ tumors. Associations between the metabolite profiles and the combined transcriptome of the xenografts and the host, reflected molecular signatures of invasion, comprising extracellular matrix degradation and reorganization, growth factor binding, and vascular remodeling.

**Conclusion:**

Integrating metabolic profiles and gene expression of highly invasive PDOX allows *in vivo* monitoring of progression in the non-enhancing tumor infiltration zone and provides insights into the remodeling of the extracellular matrix that is essential for cell-cell communication and regulation of cellular processes. The changes of the structural and biochemical properties of the extracellular matrix are of importance for the biological behavior of the tumors and may be subjected to therapeutic targeting.

